# The Association between Chronic Hemodialysis and Toe Pinch Force in Japanese Patients: Cross-Sectional Study

**DOI:** 10.3390/healthcare9121745

**Published:** 2021-12-17

**Authors:** Hiroaki Kataoka, Nobuyuki Miyatake, Naoko Matsuda, Yasuaki Hikasa, Naomi Kitayama, Shion Nagai, Satoshi Tanaka

**Affiliations:** 1 Department of Physical Therapy, Faculty of Health Sciences, Okayama Healthcare Professional University, Okayama 700-0913, Japan; 2 Department of Hygiene, Faculty of Medicine, Kagawa University, Takamatsu 760-0016, Japan; miyatake.nobuyuki@kagawa-u.ac.jp; 3 Department of Rehabilitation, Osafune Clinic, Okayama 701-4264, Japan; n.kiyose@osafune-clinic.com; 4 Department of Rehabilitation, Obata Medical Clinic, Okayama 708-0806, Japan; szknooyyy@gmail.com; 5 Rehabilitation Center, KKR Takamatsu Hospital, Takamatsu 760-0018, Japan; quincy.archibald.12@gmail.com (N.K.); slmdpt.22@gmail.com (S.N.); 6 Department of Physical Therapy, Faculty of Health and Welfare, Prefectural University of Hiroshima, Hiroshima 723-0053, Japan; s-tanaka@pu-hiroshima.ac.jp

**Keywords:** chronic hemodialysis, type 2 diabetes mellitus, toe pinch force

## Abstract

The purpose of this cross-sectional study was to investigate the effect of chronic hemodialysis on toe pinch force (TPF). A total of 37 chronic hemodialysis patients without type 2 diabetes mellitus (T2DM) (age: 69.4 ± 11.8 years, duration of hemodialysis: 3.5 ± 3.4 years) were enrolled in this study. The TPF in chronic hemodialysis patients without T2DM was compared with that in 34 apparently healthy participants and 37 chronic hemodialysis patients with T2DM. There was no significant difference in clinical profiles between healthy participants and chronic hemodialysis patients with and without T2DM. The TPF in chronic hemodialysis patients without T2DM was lower compared with that in healthy participants (2.70 ± 1.05 kg vs. 3.34 ± 0.99 kg, *p* = 0.025). In addition, the TPF in patients with T2DM was even lower compared with that in patients without T2DM (2.12 ± 1.01 kg vs. 2.70 ± 1.05 kg, *p* = 0.042). This study showed a dramatic reduction in TPF in chronic hemodialysis patients, especially in those with T2DM.

## 1. Introduction

The number of dialysis patients in Japan has exceeded 340,000; most of these dialysis patients are hemodialysis patients, and diabetic nephropathy is the most frequent cause of the introduction of hemodialysis [1]. The number of dialysis patients has been increasing steadily as advancements in dialysis therapy have led to an increase in the number of patients on long-term dialysis. To meet the current demand for improved activities of daily living (ADL) performance and quality of life (QOL) in patients receiving medical care, appropriate treatment aiming to avoid any degradation in ADL performance and to ensure that even hemodialysis patients are capable of performing the same ADL as those performed by healthy individuals is needed.

Physical activity was reported to be lower in hemodialysis patients on the day of dialysis compared with that in the general population [2], and motor function was found to be reduced by as much as 50% especially in elderly patients on hemodialysis [3]. In particular, the exercise tolerance in hemodialysis patients has been reported to decrease to the same level as that in patients with heart failure and/or chronic obstructive pulmonary disease [4]. Previous studies have suggested that exercise therapy may be associated with a favorable vital prognosis [5]. Therefore, exercise therapy is recommended for hemodialysis patients to maintain or improve vital prognosis, motor function, exercise tolerance, ADL, and QOL. Motor dysfunctions in hemodialysis patients are frequently caused by skeletal muscle weakness associated with reduced physical performance. Previous studies revealed that the lower limb muscle strength in hemodialysis patients reduced to approximately half of that in healthy individuals matched for sex and age [3,6,7]. Generally, lower limb muscle weakness is assumed to be associated with reduced physical performance, such as walking ability and balance. The toes also play an important role in gait function (e.g., stabilization of walking pattern and balance control in the gait) [8,9,10]; reduced toe pinch force (TPF) has been reported to increase the risk of falls during walking [11,12]. Aerobic exercise, especially walking exercise, is positively recommended for hemodialysis patients; therefore, it is crucial to assess TPF in these patients. However, TPF is infrequently assessed in daily clinical practice. Furthermore, there have been no studies investigating TPF in these patients to date. Based on previous reports [3,6,8], we believe that the evaluation of TPF in routine clinical practice is useful because the TPF in hemodialysis patients may decrease with time.

Therefore, the purpose of this cross-sectional study was to investigate the hypothesis that chronic hemodialysis patients have reduced TPF.

## 2. Materials and Methods

### 2.1. Study Population

A total of 37 chronic hemodialysis patients without type 2 diabetes mellitus (T2DM) were included in this cross-sectional study. The TPF in chronic hemodialysis patients without T2DM was compared with that in 34 healthy participants and 37 chronic hemodialysis patients with T2DM. The study period was April to August 2021. The study population had no habit of exercise. Chronic hemodialysis patients with and without T2DM were recruited from the inpatients and outpatients in KKR Takamatsu Hospital, Osafune clinic, and Obata Medical Clinic. Controls were voluntarily recruited from healthy people living in the community. An evaluation of the foot structure was performed by a physical therapist using inspection and palpation. The exclusion criteria of this study were as follows: (1) patients with acute metabolic disorders, (2) patients with acute or chronic bone and joint disease and/or cerebral vascular disease, (3) patients with severe chronic diabetic complications, (4) patients with foot/toe deformity and/or foot edema, (5) patients with highly restricted daily activities, and/or (6) patients who seemed inappropriate for this study. The present study was approved by the Ethics Committee of Okayama Healthcare Professional University (approval number: 0030), and informed consent was obtained from all participants.

### 2.2. Clinical and Laboratory Measurements

We collected data on sex, age (years), height (cm), body weight (kg), body mass index (BMI; kg/m^2^), duration of T2DM (years), duration of hemodialysis (years), systolic blood pressure (SBP; mmHg), diastolic blood pressure (DBP; mmHg), heart rate (HR; bpm), and medications, and laboratory test results. In chronic hemodialysis patients with T2DM, the levels of fasting plasma glucose (mg/dL), glycoalbumin (%), albumin (g/dL), hemoglobin (g/dL), C-reactive protein (mg/dL), phosphorus (mg/dL), calcium (mg/dL), β2-microglobulin (mg/dL), blood urea nitrogen (mg/dL), creatinine (mg/dL), and normalized dialysis dose (Kt/Vurea) were measured using standard methods. The geriatric nutritional risk index (GNRI) was used as an indicator of nutritional status [13]. These measurements were carried out prior to hemodialysis. The diagnosis of T2DM and diabetic nephropathy was performed according to the guidelines of the Japan Diabetes Society [14].

### 2.3. Measurement of TPF

TPF was measured using the same dynamometer and testing procedures as those used in our previous study [15]. TPF was measured using a Checker-kun (Nisshin Sangyo Inc., Saitama, Japan). Participants sat on a chair, crossing their arms over their chest. A pinch force dynamometer was then attached to participant’s foot between the digitus primus and digitus secundus while the participant sat with 90° hip joint flexion and 90° knee joint flexion. The test was performed twice each on the left and right sides, and an average of the best results for the left and right feet was then calculated.

### 2.4. Outcome Measures

The primary outcome measure of this cross-sectional study was the effect of hemodialysis on TPF. The secondary outcome measure was the effect of diabetes on the TPF in chronic hemodialysis patients.

### 2.5. Sample Size Calculation

In our previous study [15], we assumed an effect size of 1.28 and a standard deviation of 1.81. In that study, we needed to include 65 participants to achieve an 80% confidence that the study would detect a difference in TPF at a two-sided significance level of 5%. In addition, assuming 10% of cases to be dropout cases, we finally included a total of 71 cases. Therefore, we included 34 healthy controls and 37 chronic hemodialysis patients without T2DM in the present study. Furthermore, to study the secondary outcome, the number of chronic hemodialysis patients with T2DM needed to be the same as that of chronic hemodialysis patients without T2DM.

### 2.6. Statistical Analysis

The data are expressed as mean ± standard deviation (SD). Variables were compared among the three groups using the one-way analysis of variance and Tukey method. The comparisons of variables between any two groups were performed using an unpaired *t*-test and χ^2^ test. A *p* value of <0.05 was considered statistically significant. All analyses were performed using JMP statistical software version 12.1.0 (SAS Institute, Cary, NC, USA).

## 3. Results

**Figure 1:**
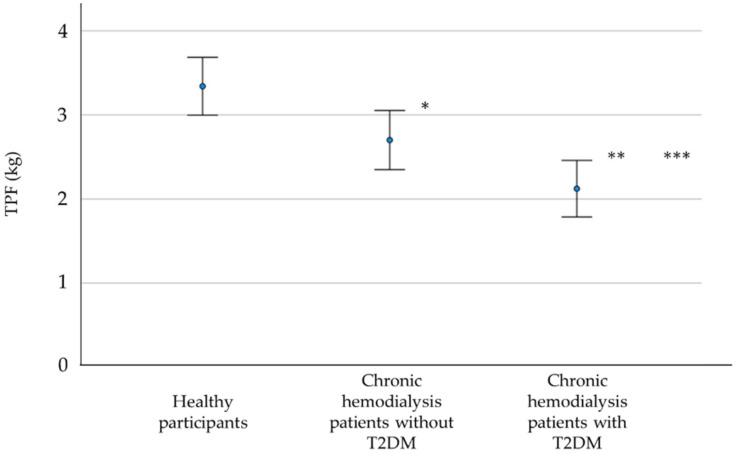
Comparison of toe pinch force (TPF) among healthy participants and chronic hemodialysis patients with and without type 2 diabetes mellitus (T2DM). Healthy participants vs. Chronic hemodialysis patients without T2DM, * *p* = 0.025; Chronic hemodialysis patients without T2DM vs. Chronic hemodialysis patients with T2DM, ** *p* = 0.042; Healthy participants vs. Chronic hemodialysis patients with T2DM, *** *p* < 0.001.

The clinical profiles of all participants are summarized in Table 1. There were no significant differences in terms of sex, age, height, body weight, BMI, SBP, DBP, and HR between healthy participants and chronic hemodialysis patients with and without T2DM. The comparison of TPF among the three groups is shown in Figure 1. The TPF in chronic hemodialysis patients without T2DM was significantly lower than that in healthy controls (2.70 ± 1.05 kg vs. 3.34 ± 0.99 kg, *p* = 0.025). The TPF in chronic hemodialysis patients with T2DM was significantly lower than that in chronic hemodialysis patients without T2DM (2.12 ± 1.01 kg vs. 2.70 ± 1.05 kg, *p* = 0.042). The TPF in chronic hemodialysis patients with T2DM was significantly lower than that in healthy participants (2.12 ± 1.01 kg vs. 3.34 ± 0.99 kg, *p* < 0.001). The TPF in chronic hemodialysis patients without T2DM was approximately 19.2% lower than that in healthy participants. The TPF in chronic hemodialysis patients with T2DM was approximately 21.4% lower than that in chronic hemodialysis patients without T2DM.

## 4. Discussion

In this study, we aimed to investigate the effect of chronic hemodialysis on TPF. The results showed that the TPF in chronic hemodialysis patients without T2DM was significantly lower compared with that in healthy individuals.

Previous studies on muscle strength in hemodialysis patients found reduced grip strength and lower limb muscle strength in these patients. A systematic review on grip strength in hemodialysis patients revealed that the grip strength ranged from 12–38 kg in male patients and 11–26 kg in female patients, and several hemodialysis patients showed reduced grip strength [16]. Studies investigating knee extension muscle strength in hemodialysis patients have reported muscle weakness at a rate of approximately 69% in comparison with that of healthy individuals [7,17]. In summary, numerous studies have reported reduced hand grip strength and lower limb muscle strength (mainly knee extensor muscle strength) in hemodialysis patients; however, TPF has not yet been investigated in these patients. Grip strength reflects muscular strength throughout the body. In addition, because knee extensor muscle strength reflects muscular strength in the lower limbs, previous studies focused on grip strength and knee extensor muscle strength and not TPF. The toes play important roles in posture stabilization and balance function during standing and walking and in generating forward propulsive forces during walking [8,9,10]. In fact, aging is known to reduce TPF [18,19], leading to an increased risk of falls [11,12]. Previous studies have reported a fall incidence of 27–55% in hemodialysis patients [20,21,22,23], and fractures caused by falls markedly reduce patients’ ADL and QOL. In particular, the risk of fractures in hemodialysis patients is higher than that in the general population because these patients, on average, are younger at the time of fracture than the general population [24]; the incidence of proximal femur fractures in these patients is approximately fivefold that in the general population [25]. Therefore, we believe that the TPF in hemodialysis patients should always be evaluated before an exercise intervention to provide safe and effective motor therapy. This study shows that the TPF in chronic hemodialysis patients is lower than that in healthy individuals, and, to the best of our knowledge, this is a new finding on the effect of hemodialysis on lower limb muscle strength. In addition, the results of this study suggest the necessity of toe resistance training in chronic hemodialysis patients. We have previously reported the efficacy of toe resistance training in patients with T2DM. We believe that this resistance training is suitable for chronic hemodialysis patients because it is low-intensity, safe, and effective in a short period of time. We are currently conducting an intervention study in chronic hemodialysis patients to verify the effectiveness of toe resistance training.

Sarcopenia occurs more frequently in hemodialysis patients than in the general population [26]. According to previous studies, approximately 20% of these patients already have sarcopenia at the age when hemodialysis is introduced [27], and 33% of such patients older than 60 years have sarcopenia [28]. Sarcopenia is mainly caused by the reduction of muscle quantity and muscle force due to aging, which may also be related to the reduction of TPF. In addition, this may lead to a decrease in TPF because dialysis treatment leads to progressive muscle protein catabolism and marked skeletal muscle atrophy [29]. Moreover, hemodialysis treatment has been shown to be associated with prolonged restraint time, which makes it more likely to cause reduced physical activity due to post-treatment fatigue, and muscle weakness may be caused by metabolic disorders associated with renal failure and dietary restriction, which may lead to poor nutritional status [7]. In this study, nutritional status was assessed using the GNRI; and we observed that 80% participants were at “no risk of nutritional impairment” or “at risk of mild nutritional impairment”. The lack of exercise habits rather than nutritional disturbance may be the factor responsible for toe muscle deterioration since this study included patients that lacked such habits.

We also found that the TPF in chronic hemodialysis patients with T2DM was even lower than that in chronic hemodialysis patients without T2DM. Previously, we compared TPF between patients with T2DM and healthy individuals, and our findings showed that the TPF in patients with T2DM was significantly lower than that in healthy individuals, indicating that diabetes is strongly associated with TPF reduction [15]. In addition, a previous study investigating TPF in patients with diabetic nephropathy of stages 1 to 3 has shown that diabetic nephropathy is strongly associated with toe muscle weakness [30]. Thus, the further decrease in TPF in chronic hemodialysis patients with T2DM in this study is expected. Possible mechanisms by which diabetes and diabetic nephropathy may reduce TPF, including the atrophy of muscle fibers caused by hyperglycemia [31], increased protein catabolism in skeletal muscle [32], the accumulation of urea in the blood and skeletal muscle due to reduced renal function [33], skeletal muscle adiposity [34], and increased intra-skeletal fat [35], have been suggested.

In this study, TPF was defined as the force required to pinch the hallux and index toe together. The intrinsic muscles of the foot, such as the adductor hallucis, plantar interossei, dorsal interosseous, flexor hallucis brevis, flexor digitorum brevis, and lumbrical muscles, are primarily involved in such movement of the flanking toes. The atrophy of these intrinsic muscles reduces TPF and inhibits flexion and extension movements of the metatarsophalangeal joint. Therefore, the dominance of external muscle activity results in an extension moment at the metatarsophalangeal joint, and a large flexion moment at the interphalangeal joint can deform the toes, eventually leading to diabetic foot lesions.

This study has certain limitations. First, this was a cross-sectional study; therefore, a longitudinal study needs to be performed to clearly identify the factors reducing TPF in chronic hemodialysis patients. Second, this study included patients with only T2DM. It is unclear whether similar results can be obtained in patients with type 1 diabetes. Third, diabetic polyneuropathy (DPN) has been reported as a factor associated with reduced TPF [15], but DPN has not been evaluated. Patients with severe chronic complications were excluded from this study, but it cannot be ruled out that patients with mild DPN may also have a reduced TPF as DPN generally emerges as a chronic complication at the early stage of diabetes. Fourth, TPF is thought to be closely related to muscle mass, but muscle mass was not measured in this study. In the future, muscle mass should be assessed and its relationship with TPF should be further investigated. The results obtained in this study warrant further investigation into the efficacy of physiotherapy to treat reduced TPF in chronic hemodialysis patients with and without T2DM.

## 5. Conclusions

We found reduced TPF in chronic hemodialysis patients, especially in those with T2DM.

## Figures and Tables

**Table 1:** Clinical characteristics of healthy participants and chronic hemodialysis patients with and without type 2 diabetes mellitus (T2DM).

	Healthy Participants	Chronic Hemodialysis Patients without T2DM	Chronic Hemodialysis Patients with T2DM	*p* Value
n	34	37	37	
Sex (male, n)	25	25	27	0.790
Age (years)	67.8 ± 7.3	69.4 ± 11.8	69.7 ± 7.3	0.887
Height (cm)	166.4 ± 7.1	163.3 ± 8.6	163.3 ± 7.3	0.993
Body weight (kg)	69.6 ± 10.6	64.9 ± 14.0	66.3 ± 9.4	0.611
BMI (kg/m^2^)	25.0 ± 2.9	24.2 ± 3.7	24.8 ± 2.7	0.383
SBP (mmHg)	136.9 ± 15.2	138.9 ± 25.8	135.1 ± 14.5	0.439
DBP (mmHg)	73.0 ± 13.6	74.2 ± 12.7	71.1 ± 11.5	0.283
HR (bpm)	68.7 ± 10.3	68.9 ± 11.9	68.0 ± 10.5	0.710
Duration of hemodialysis (years)		3.5 ± 3.4	3.9 ± 3.8	0.630
GNRI score				
No risk, n (%)		19 (51.3)	24 (64.9)	0.328
Mild risk, n (%)		11 (29.7)	10 (27.0)	
Moderate risk, n (%)		7 (19.0)	3 (8.1)	
Albumin (g/dL)		3.6 ± 0.3	3.6 ± 0.3	0.668
Hemoglobin (g/dL)		11.3 ± 1.6	11.3 ± 1.3	0.844
CRP (mg/dL)		0.4 ± 0.4	0.3 ± 0.3	0.263
Phosphorus (mg/dL)		5.6 ± 1.2	5.1 ± 1.2	0.101
Calcium (mg/dL)		8.5 ± 0.7	8.6 ± 0.7	0.567
β2MG (mg/dL)		23.9 ± 5.7	21.9 ± 5.1	0.110
BUN (mg/dL)		61.9 ± 2.3	55.7 ± 2.0	0.054
CRE (mg/dL)		9.4 ± 2.6	8.8 ± 2.4	0.347
eGFR (mL/min/1.73m^2^)		4.4 ± 2.0	5.0 ± 1.6	0.136
Normalized dialysis dose (Kt/V)		1.4 ± 0.3	1.4 ± 0.2	0.714
FPG (mg/dL)			166.8 ± 55.4	
Glycoalbumin (%)			20.8 ± 4.4	
Duration of diabetes (years)			16.9 ± 10.5	
Medication				
Insulin, n (%)			7 (20.0)	
OHA, n (%)			9 (24.3)	
Insulin + OHA, n (%)			10 (27.0)	
None, n (%)			11 (29.7)	

Data are presented as the mean ± standard deviation. T2DM, type 2 diabetes mellitus; BMI, body mass index; SBP, systolic blood pressure; DBP, diastolic blood pressure; HR, heart rate; GNRI, geriatric nutritional risk index; CRP, C-reactive protein; β2MG, β2-microglobulin; BUN, blood urea nitrogen; CRE, creatinine; eGFR, estimated glomerular filtration rate; FPG, fasting plasma glucose; OHA, oral hypoglycemic agent.

## Data Availability

Not applicable.
